# Characterization of miR-34a-Induced Epithelial–Mesenchymal Transition in Non-Small Lung Cancer Cells Focusing on p53

**DOI:** 10.3390/biom11121853

**Published:** 2021-12-09

**Authors:** Masashi Kawami, Shinnosuke Takenaka, Mizuki Akai, Ryoko Yumoto, Mikihisa Takano

**Affiliations:** Department of Pharmaceutics and Therapeutics, Graduate School of Biomedical and Health Sciences, Hiroshima University, 1-2-3 Kasumi, Minami-ku, Hiroshima 734-8553, Japan; m205991@hiroshima-u.ac.jp (S.T.); b184961@hiroshima-u.ac.jp (M.A.); ryumoto@hiroshima-u.ac.jp (R.Y.); takanom@hiroshima-u.ac.jp (M.T.)

**Keywords:** epithelial–mesenchymal transition, microRNAs, miR-34a, non-small lung cell cancer cells, p53

## Abstract

Background: Epithelial–mesenchymal transition (EMT), a phenotypic conversion of the epithelial to mesenchymal state, contributes to cancer progression. Currently, several microRNAs (miRNAs) are associated with EMT-mediated cancer progression, but the contribution of miR-34a to EMT in cancer cells remains controversial. The present study aimed to clarify the role of miR-34a in the EMT-related phenotypes of human non-small cell lung cancer (NSCLC) cell lines, A549 (p53 wild-type) and H1299 (p53-deficient). Methods: The miR-34a mimic and p53 small interfering RNA (siRNA) were transfected into the cells using Lipofectamine, and the obtained total RNA and cell lysates were used for real-time polymerase chain reaction and Western blotting analysis, respectively. Results: The introduction of the miR-34a mimic led to an increase in the mRNA and protein expression levels of α-smooth muscle actin (*α-SMA*), a mesenchymal marker gene, in A549, but not in H1299 cells. Additionally, miR-34a-induced the upregulation of p53 activity and migration was observed in A549, but not in H1299 cells. However, under the p53-knockdown condition, only α-SMA upregulation by miR-34a was abolished. Conclusion: These findings indicate a close relationship between p53 and miR-34a-induced EMT in p53-wild type NSCLC cells, which provides novel insights about the role of miR-34a in EMT-like phenotypic changes in NSCLC.

## 1. Introduction

MicroRNAs (miRNAs) are a class of non-coding RNAs that function as master regulators of the genome by modulating the expression of tens to hundreds of genes and controlling several cellular pathways at once. Downregulation of certain miRNAs is frequently observed in a variety of cancers, which leads to poor prognosis. For example, it has been reported that reduced let-7 expression corresponds to shorter survival in patients with lung cancers [1]. Thus, there is currently great interest in the relationship between miRNAs and the pathogenesis of several diseases. In fact, the number of miRNAs that are considered as biomarkers and therapeutic agents for several diseases such as tumors [2] and organ fibrosis [3] are increasing. In particular, miR-34a-5p is a leading miRNA that shows an antitumor effect in preclinical and clinical studies [4,5]. Numerous studies have revealed that the tumor-suppressing effect of miR-34a is correlated with miR-34a-mediated downregulation of cell cycle proteins, such as cyclin-dependent kinase 4/6, anti-apoptosis proteins, such as B-cell chronic lymphocytic leukemia (CLL)/lymphoma 2, and metastasis-related proteins, such as c-Myc and cluster of differentiation (CD) 44 [6]. Thus, miR-34a is one of the most familiar miRNAs with anti-tumor effects.

Epithelial–mesenchymal transition (EMT) is a biological process in which epithelial cells downregulate epithelial characteristics and acquire a mesenchymal state, which was first proposed and characterized by Elizabeth Hay [7]. During EMT, epithelial cells lose polarity and cobblestone morphology, while acquiring a spindle-shaped morphology with an increase in mesenchymal marker genes such as fibronectin, α-smooth muscle actin (α-SMA), and vimentin. There is currently a consensus that the EMT process is closely related to the malignant progression of carcinoma cells, including tumor-initiating properties, motility, the ability to disseminate and elevated resistance to killing by commonly employed chemotherapeutics [8]. Thus, EMT is now considered a targetable event to prevent the initiation and progression of tumors.

So far, we have demonstrated that several miRNAs (miR-34a, -222 and -484) are associated with certain drug-induced EMT in alveolar epithelial cell lines derived from humans and rats [9,10,11,12], which assumes that serious lung injury triggered by the drugs is linked with the conversion of epithelial to mesenchymal state in the cells through the EMT process. In these reports, miR-34a confers EMT-like phenotypes in two modified type II alveolar epithelial cell lines, rat-derived RLE/Abca3 and human-derived A549/ABCA3 cells, as evidenced by the upregulation of α-SMA, a representative mesenchymal marker gene, in miR-34a mimic-transfected cells [9,10]. Other research groups have reported that miR-34a promotes EMT in human intrahepatic biliary epithelial cells via upregulation of phosphorylated SMAD2/3, a popular EMT-inducing signaling pathway [13]. Conversely, miR-34a is also known as an EMT-suppressing miRNA that inhibits EMT-inducing transcription factors (EMT-TFs), including SNAIL, SLUG, zinc finger E-box binding protein (ZEB) 1, ZEB2, and TWIST1 [14,15]. Thus, the mechanism by which miR-34a contributes to the EMT remains controversial. As EMT corresponds to cancer progression, the net contribution of miR-34a to EMT should be urgently determined prior to the clinical use of miR-34a preparations in the near future.

As miR-34a was first discovered as a downstream gene of p53, a tumor suppressor gene [16], p53 functions as a transcriptional factor for miR-34a. Although our previous report demonstrated that drug-induced EMT is associated with the p53-miR-34a axis in A549/ABCA3 cells [10], there are only a few studies focusing on the involvement of the p53-miR-34a axis in the EMT process. We accumulated EMT-related information using the non-small cell lung cancer (NSCLC) cell line, A549, with wild-type p53. Conversely, H1299, another NSCLC cell line, is genetically p53 deficient. Accordingly, the present study aimed to compare miR-34a-induced phenotypic changes between miR-34a-overexpressed A549 and H1299 cells and clarify the role of p53 in miR-34a-induced EMT in NSCLC cells.

## 2. Materials and Methods

### 2.1. Cell Lines and Cell Culture

Human NSCLC cell lines, A549 and H1299, were obtained from the RIKEN Bioresource Center and American Type Culture Collection, respectively. These cells were cultured in the Dulbecco’s modified Eagle’s medium containing 10% fetal bovine serum, 100 IU/mL penicillin, and 100 mg/mL streptomycin in an atmosphere of 5% carbon dioxide (CO_2_)/95% air at 37 °C, and sub-cultured every 7 d using 0.25% trypsin and 1 mM ethylenediaminetetraacetic acid (EDTA). Fresh medium was replaced every 2–3 d.

### 2.2. Transfection with the miR-34a Mimic or siRNA for p53

A549 or H1299 cells grown on 12-well plates were transfected with 20 pmol/well of miRIDLAN microRNA miR-34a-5p-mimic or miRIDLAN microRNA mimic negative control (GE Healthcare Japan, Tokyo, Japan) using Lipofectamine^TM^2000 (1 µL/well; Thermo Fisher Scientific Inc., Waltham, MA, USA) for 24 h.

Similarly, A549 or H1299 cells grown on 12-well plates were transfected with 10 pmol/well of siRNA for p53 or MISSION^®^ siRNA Universal Negative Control (siCont.) (Merck KGaA, Darmstadt, Germany) for 24 h. The sequences of investigated siRNAs for p53 (#1–3) are listed in Figure S1A. All the siRNAs clearly downregulated the protein expression levels of p53 (Figure S1B), and significantly decreased the mRNA expression of cyclin-dependent kinase inhibitor 1A (CDKN1A), a transcriptional target of p53 (Figure S1C). As siRNA #1 showed the most potent inhibitory effect on the mRNA expression of CDKN1A, it was selected as the siRNA for p53 for subsequent experiments.

### 2.3. Apoptosis Assay

After transfection in the 12-well plate for 24 h, a further 24 or 72 h culture with fresh medium without transfection reagents was performed. The cells were then washed and trypsinized. The detached cells were counted, and the cell suspension was adjusted to 1 × 10^6^ cells/mL. After 1 mL cell suspension was centrifuged at 500× *g* for 5 min, the pellet was resuspended in 85 µL binding buffer (150 mM sodium chloride (NaCl), 5 mM potassium chloride (KCl), 1.8 mM calcium chloride (CaCl_2_), 1.0 mM magnesium chloride (MgCl_2_), and 50 mM 4-(2-hydroxyethyl)-1-piperazineëthanesulfonic acid (HEPES)/sodium hydroxide (NaOH), pH 7.4). Then, fluorescein isothiocyanate (FITC)-labeled Annexin-V (10 µL) and propidium iodide (PI) (5 µL) were added to the cell suspension and incubated for 15 min at approximately 23 °C. FITC and PI fluorescence were then analyzed using Guava easyCyte^®^ (Luminex Corporation, Austin, TX, USA). The raw data was analyzed and visualized by FLOWJO^®^.

### 2.4. Scratch-Wound Assay

After transfection in a 12-well plate for 24 h, a 48 h culture with fresh medium without transfection reagents was performed. Then, a sterile 20–100 µL pipette tip was held vertically to scratch a cross in each well. The detached cells were removed by washing with fresh culture medium, and the scratch line in each well was observed and recorded by phase-contrast microscopy at 0 h. The scratch closure was monitored and imaged for 12 h, and the cell-free area was analyzed using the Image J software Ver. 1.53 m (https://imagej.nih.gov/ij/, accessed on 9 October 2021).

### 2.5. Detection of mRNA Expression Using Real-Time PCR Analysis

Total RNA was extracted from the treated cells using the Monarch Total RNA Miniprep Kit (New England Biolabs Inc., Ipswich, MA, USA), according to the manufacturer’s instructions. Total RNA was converted into complementary DNA using ReverTra Ace (Toyobo, Osaka, Japan). Real-time PCR analysis was conducted using the CFX Connect™ Real-Time PCR detection system (Bio-Rad Laboratories, Inc., Hercules, CA, USA) with Luna Universal qPCR Master Mix (New England Biolabs Inc.), according to the manufacturer’s instructions. The primer sequences used are listed in Table 1. The mRNA expression levels were normalized to that of rat glyceraldehyde-3-phosphate dehydrogenase (*GAPDH*), a housekeeping gene.

### 2.6. Detection of miRNA Expression Using Real-Time PCR Analysis

The method used to obtain total RNA was the same as that used for mRNAs. miRNA expression was evaluated as previously described [11]. The primers for the amplification of miR-34a and RNU6-2 were obtained from QIAGEN (Hilden, Germany). The expression levels of miR-34a were normalized to those of RNU6-2 in the treated cells.

### 2.7. Detection of Protein Expression Using Western Blotting Analysis

Protein expression was evaluated using Western blotting analysis, as previously described [11]. Briefly, cell lysates obtained from the treated cells were subjected to sodium dodecyl sulfate-polyacrylamide gel electrophoresis (SDS-PAGE) using 10% polyacrylamide gels and transferred onto polyvinylidene difluoride (PVDF) membranes. The membranes were blocked with 0.1% skim milk, and subsequently incubated for 2 h with the primary antibodies against α-SMA protein (1:1000 dilution; GeneTex, San Antonio, TX, USA), p-SMAD2 protein (1:2000 dilution; Signalway Antibody Co., Ltd., College Park, ML, USA), p53 protein (1:2000 dilution; Merck, Darmstadt, Germany), and β-actin (1:2000 dilution; GeneTex). The membranes were washed three times in Tris-buffered saline-Tween (TBS-T; 20.5 mM Tris, 150 mM NaCl, 0.05% Tween 20, pH 7.5), and then incubated for 1 h with horseradish peroxidase (HRP)-labeled secondary antibodies (1:5000 dilution; GE Healthcare, Milwaukee, WI, USA). After washing thrice with TBS-T, the antibody complexes were visualized using Immobilon Forte Western HRP reagent (Merck). The detection of chemiluminescence intensity was performed using FUSION^®^ (Vilber, Collégien, France).

### 2.8. Statistical Analysis

Data are expressed as the mean ± standard error of the mean (S.E.M). Statistical analysis was performed using one-way analysis of ANOVA followed by Tukey’s test for multiple comparisons. Statistical significance was set at *p* < 0.05.

## 3. Results

### 3.1. Effect of miR-34a Overexpression on the Apoptosis Ratio of A549 and H1299 Cells

**Figure 1:**
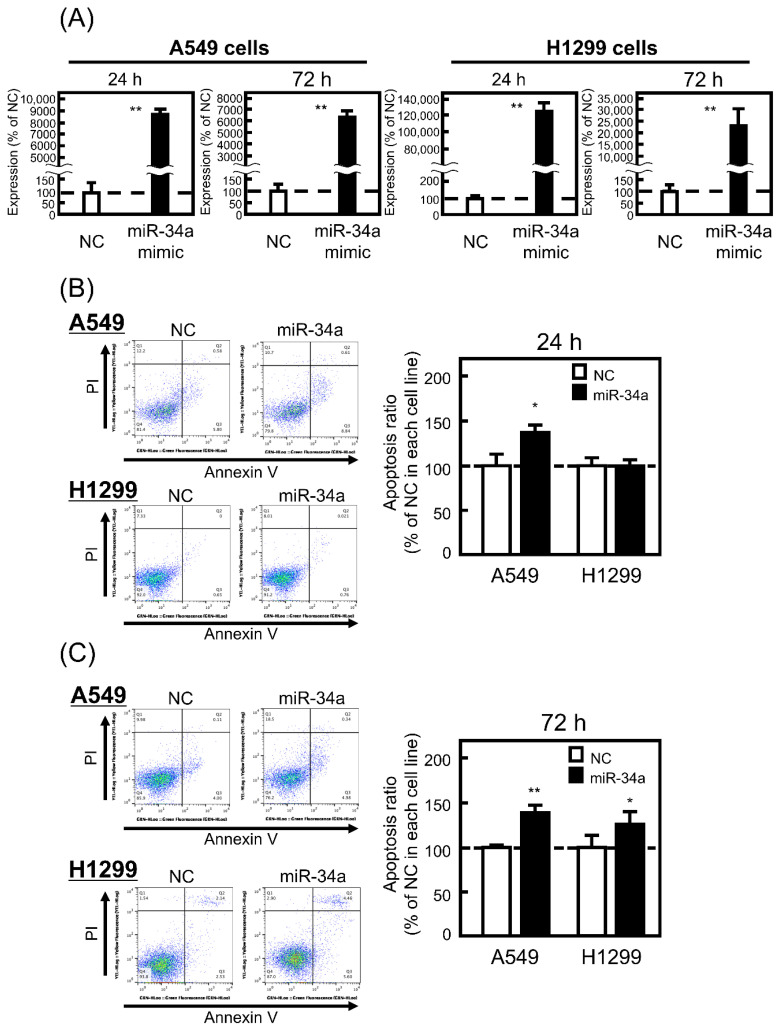
Effect of microRNA (miR)-34a overexpression on the apoptosis of A549 and H1299 cells. These cells were transfected with the negative control (NC; 20 pmol/well) or miR-34a mimic (20 pmol/well) for 24 h. After 24 or 72 h, (**A**) miR-34a expression levels were measured by real-time polymerase chain reaction (PCR) and (**B**,**C**) the ratio of apoptotic cells (annexin V-positive, propidium iodide (PI)-negative) was detected and analyzed by flow cytometry as described in Section 2. Each value represents the mean ± standard error of the mean (S.E.M). (n = 3). * *p* < 0.05, ** *p* < 0.01, significantly different from NC.

It is reported that miR-34a induces apoptosis in p53-wild type HCT116 colon cancer cell lines, but not in p53-inactivated HCT116 cells by homologous recombination [17]. Therefore, we first confirmed the effect of miR-34a overexpression on the apoptosis ratio of p53-wild type A549 and p53-deficient H1299 cells. As shown in Figure 1A, the introduction of miR-34a into both cell lines was successfully performed with high expression levels of miR-34a at both 24 and 72 h. Under the current conditions, the miR-34a mimic significantly increased the apoptosis ratio of A549 cells at 24 and 72 h, whereas miR-34a-induced apoptosis in H1299 cells was less observed compared with that in A549 cells (Figure 1B,C). These findings suggest that the p53-miR-34a axis contributes to apoptosis induction and work in A549 cells, but not in H1299 cells, as expected.

### 3.2. Effect of miR-34a Overexpression on EMT-Related Phenotypes in A549 and H1299 Cells

**Figure 2:**
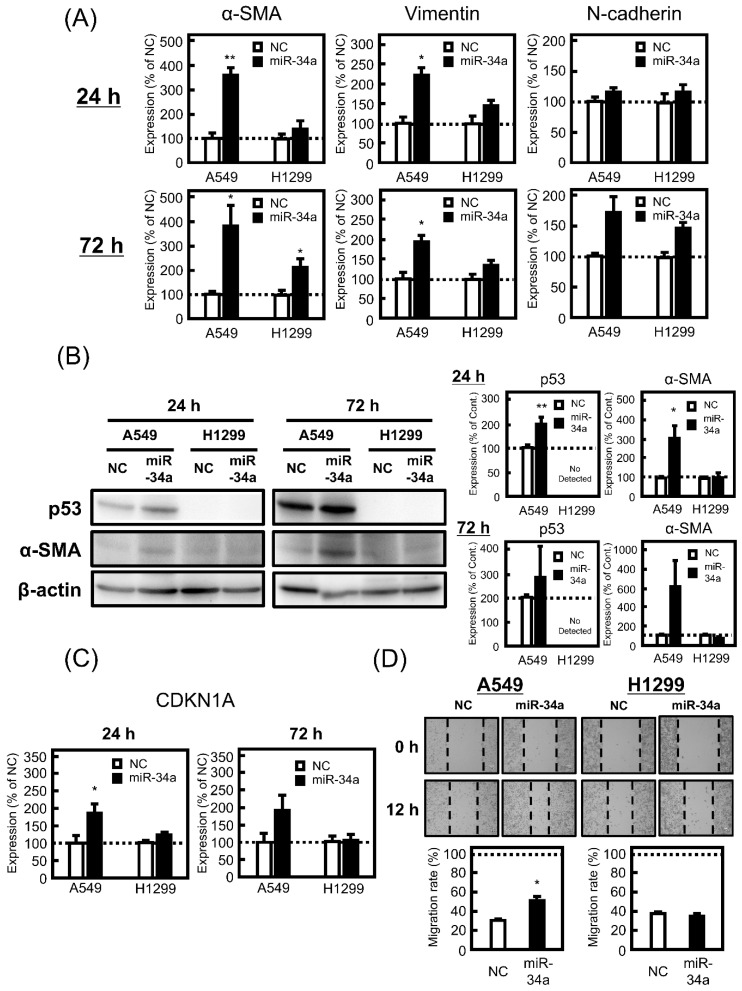
Effect of miR-34a overexpression on epithelial–mesenchymal transition (EMT)-related phenotypes in A549 and H1299 cells. These cells were transfected with NC (20 pmol/well) or miR-34a mimic (20 pmol/well) for 24 h. After 24 or 72 h, the mRNA expression levels of (**A**) α-smooth muscle actin (α-SMA), vimentin, N-cadherin, and (**C**) cyclin-dependent kinase inhibitor 1A (CDKN1A) were measured by real-time PCR. (**B**) Protein levels of α-SMA and p53 were analyzed by Western blotting. (**D**) The migration ability was confirmed by scratch-wound assay as described in Section 2. Each value represents the mean ± S.E.M. (n = 3). * *p* < 0.05, ** *p* < 0.01, significantly different from NC.

Under the same conditions as the apoptosis assay, the effect of miR-34a overexpression on EMT-like phenotypes in A549 and H1299 cells was examined. First, the effect of miR-34a on the morphology of both cell lines was confirmed, resulting in miR-34a-induced morphological changes in A549 cells, but not in H1299 cells, was observed at 24 and 72 h (Figure S2). In guidelines for research on EMT, several mesenchymal markers, such as α-SMA, N-cadherin, and vimentin are recommended as EMT indicators [18]. Among these genes, mRNA expression levels of α-SMA and vimentin were upregulated by miR-34a mimic treatment in A549 cells, but not in H1299 cells (Figure 2A), and the trend of protein expression level of α-SMA in both cells was similar to that of mRNA. Furthermore, the miR-34a mimic increased the protein expression level of p53 in A549 cells, while there was no expression of p53 in H1299 cells (Figure 2B). CDKN1A encoding p21, a transcriptional target of p53, was also upregulated by miR-34a mimic transfection in A549 cells, but not in H1299 cells (Figure 2C), which was comparable to the results for α-SMA. As shown in Figure 2D, the wound scratch assay revealed that miR-34a augmented the migration of A549 cells, but not in H1299 cells. These findings indicate a positive correlation between EMT-like phenotypes and p53 activity.

### 3.3. Effect of miR-34a Overexpression on the mRNA Expression Levels of EMT-Related Transcriptional Factors in A549 and H1299 Cells

**Figure 3:**
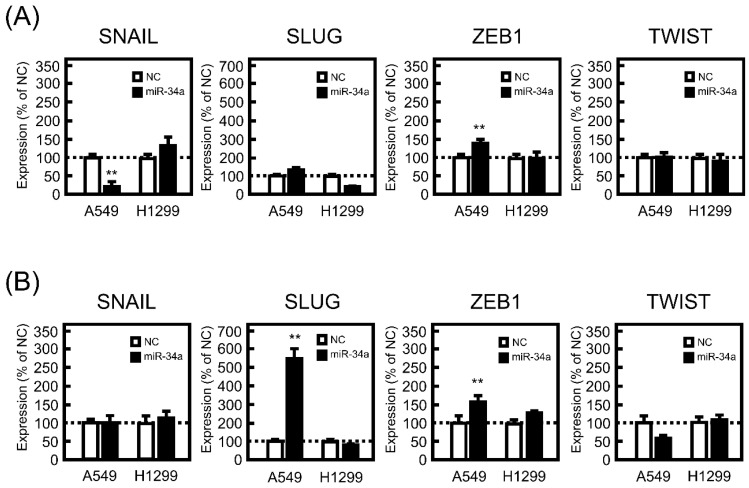
Effect of miR-34a overexpression on EMT-related transcriptional factors in A549 and H1299 cells. These cells were transfected with NC (20 pmol/well) or miR-34a mimic (20 pmol/well) for 24 h. After 24 (**A**) or 72 h (**B**), the mRNA expression levels of SNAIL, SLUG, zinc finger E-box binding protein (ZEB)-1, and TWIST were measured by real-time PCR. Each value represents the mean ± S.E.M. (n = 3). ** *p* < 0.01, significantly different from NC.

There are several EMT-related transcriptional factors, such as SNAIL, SLUG, ZEB1, and TWIST. These transcriptional factors contribute to the promotion of EMT by positively or negatively regulating the transcription of several EMT-marker genes. Therefore, the effect of miR-34a mimic transfection on the mRNA expression levels of the transcriptional factors was examined. After 24 h of treatment with the miR-34a mimic, significant decreases and increases in mRNA expression levels of SNAIL and ZEB1 were observed in A549 cells (Figure 3A). As shown in Figure 3B, a drastic increase in mRNA expression of SLUG was detected 72 h after miR-34a mimic introduction into A549 cells. Conversely, miR-34a mimic transfection had no effect on the mRNA expression levels of these transcriptional factors at either 24 or 72 h in H1299 cells (Figure 3).

### 3.4. Effect of miR-34a Overexpression on EMT-Related Phenotypes under p53-Knockdown in A549 Cells

**Figure 4:**
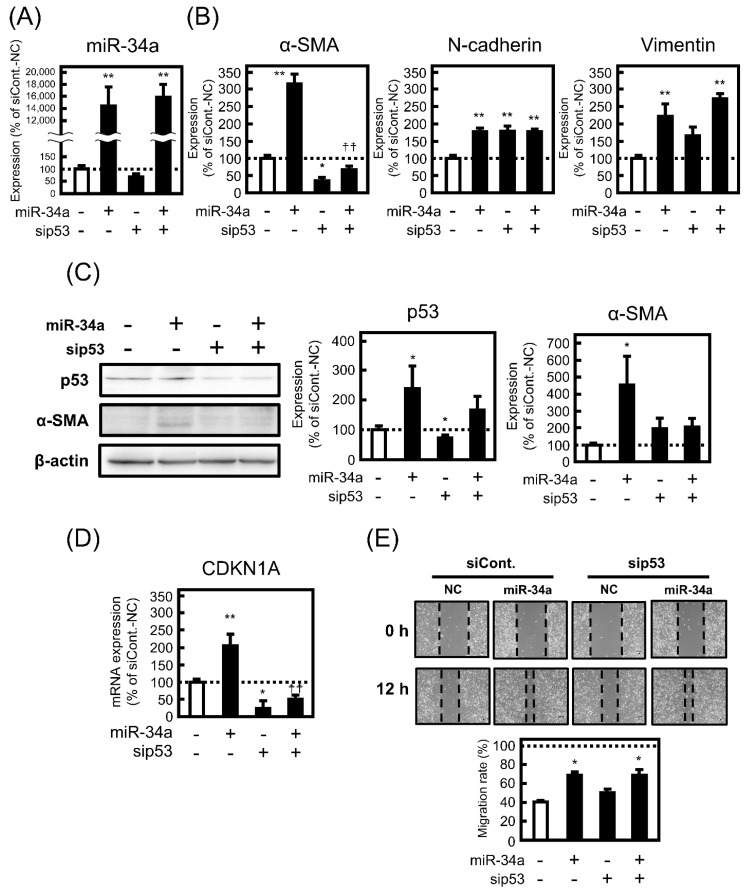
Contribution of p53 to EMT-related phenotypes in A549 cells under miR-34a overexpression. The cells were transfected with NC (20 pmol/well) or miR-34a mimic (20 pmol/well) under the control siRNA (siCont.; 10 pmol/well) or sip53 (10 pmol/well) for 24 h. After 72 h, (**A**) miR-34a and mRNA expression levels of (**B**) α-SMA, vimentin, N-cadherin, and (**D**) CDKN1A were measured by real-time PCR. (**C**) The protein levels of α-SMA and p53 were analyzed by Western blotting. (**E**) The migration ability was confirmed by scratch-wound assay as described in the Materials and methods section. Each value represents the mean ± S.E.M. (n = 3). * *p* < 0.05, ** *p* < 0.01, significantly different from NC under siCont. condition, ^††^ *p* < 0.01, significantly different from miR-34a mimic under siCont. condition.

To confirm whether p53 contributes to EMT-like phenotypic changes induced by miR-43a mimic, the effect of miR-34a overexpression for 72 h on EMT-related phenotypes in p53-knockdown A549 cells was examined. When p53 was knocked down in A549 cells, miR-34a mimic transfection was successfully conducted, as shown in Figure 4A. Notably, knockdown of p53 negated the mRNA expression level of α-SMA, but not N-cadherin and vimentin, enhanced by miR-34a mimic transfection (Figure 4B). As shown in Figure 4C, the knocking effect of sip53 was maintained in miR-34-transfected cells, by observing a slight decrease in p53 protein expression by miR-34a mimic under p53-knockdown conditions, and miR-34a-inudced upregulation of α-SMA protein expression was inhibited in p53-knockdown cells in parallel with the case of miRNA expression. A decrease in mRNA expression level of CDKN1A, the transcriptional activity of p53, was also observed in miR-34a-overexpressed cells (Figure 4D). Compared with the case of 72 h treatment, almost similar trends in the results of miR-34a transfection for 24 h were obtained (Figure S3). However, miR-34a-induced enhancement of migration rate was not reversed in p53-knockdown cells (Figure 4E).

**Figure 5:**
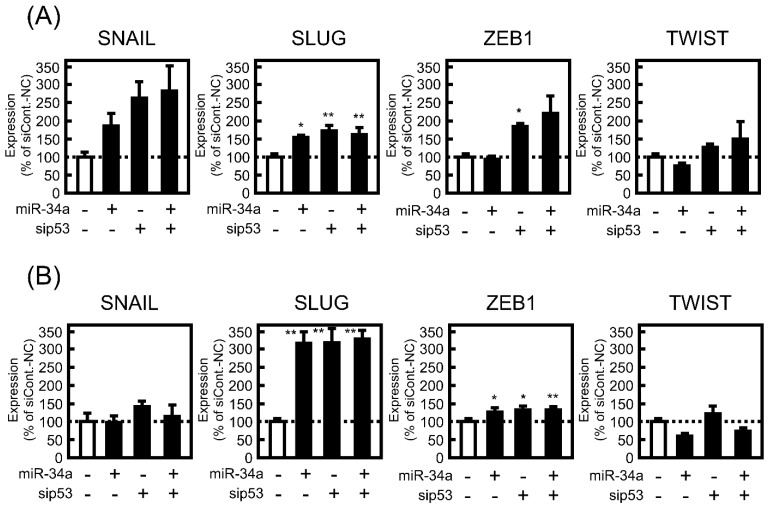
Contribution of p53 to EMT-related transcriptional factors in A549 cells under miR-34a overexpression. The cells were transfected with NC (20 pmol/well) or miR-34a mimic (20 pmol/well) under the control siRNA (siCont.; 10 pmol/well) or sip53 (10 pmol/well) for 24 h. After 24 (**A**) or 72 h (**B**), the mRNA expression levels of SNAIL, SLUG, ZEB1, and TWIST were measured by real-time PCR. Each value represents the mean ± S.E.M. (n = 3). * *p* < 0.05, ** *p* < 0.01, significantly different from miR-34a mimic under siCont. condition.

Additionally, the effect of miR-34a overexpression on EMT-inducing transcription factors under p53-knockdown conditions was examined. However, miR-34a-induced changes in mRNA expression levels of the transcriptional factors, especially in SLUG, were not affected by p53 knockdown (Figure 5). These observations indicated that p53 may specifically correspond to the expression level of α-SMA among EMT-related factors in A549 cells.

### 3.5. Effect of SB431542 (SB) on miR-34a-Induced EMT

**Figure 6:**
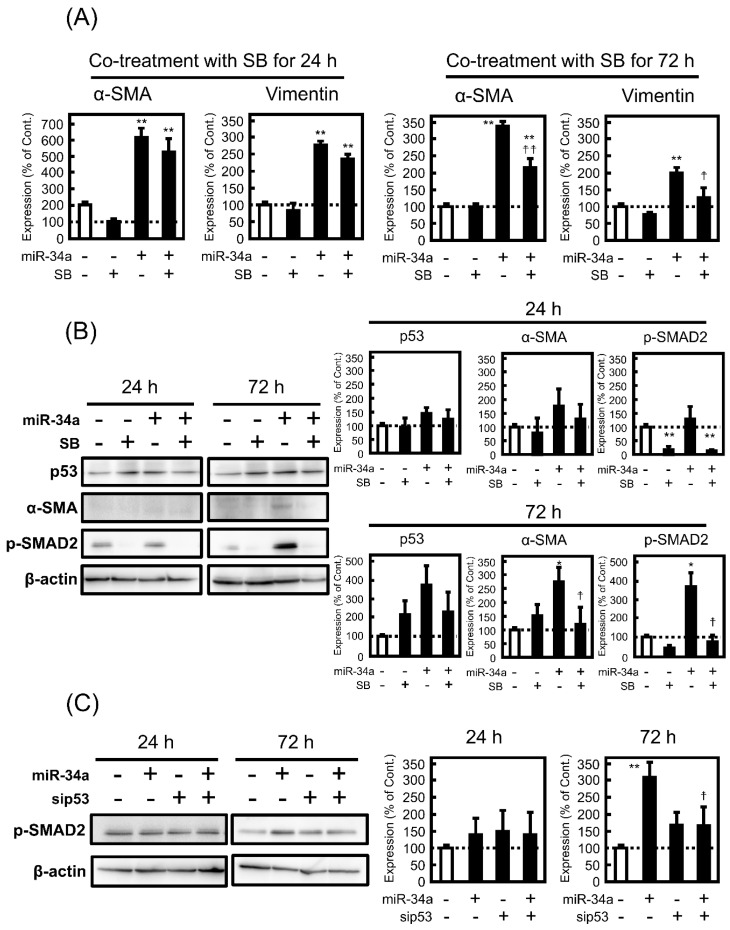
Role of the transforming growth factor (TGF)-β/SMAD signaling in miR-34a-induced changes in EMT-related phenotypes in A549 cells. The cells were transfected with NC (20 pmol/well) or miR-34a mimic (20 pmol/well) for 24 h. After removal of the medium containing the miRNA mimic, the cells were treated with or without 10 μM SB43142 (SB) for 24 or 72 h. (**A**) The mRNA expression levels of α-SMA were measured by real-time PCR. (**B**) Protein levels of α-SMA, p53, and p-SMAD2 were analyzed by Western blotting. (**C**) The cells were transfected with NC (20 pmol/well) or miR-34a mimic (20 pmol/well) under the control siRNA (siCont.; 10 pmol/well) or sip53 (10 pmol/well) for 24 h. After removal of the medium containing the miRNA mimic, the cells were treated with or without 10 μM SB43142 (SB) for 24 or 72 h. The protein levels of p-SMAD2 were analyzed by Western blotting. Each value represents the mean ± S.E.M. (n = 3). * *p* < 0.05, ** *p* < 0.01, significantly different from NC or NC under siCont. condition, ^††^ *p* < 0.01, ^†^ *p* < 0.05, significantly different from miR-34a mimic or miR-34a mimic under siCont. condition.

As miR-34a is reported to promote EMT in primary biliary cholangitis by regulating the transforming growth factor (TGF)-β1/SMAD pathway [13], the contribution of p53 to the inhibitory effect of SB, an inhibitor of the SMAD pathway, on miR-34a-induced EMT in A549 cells was examined. First, the effect of SB on the morphological changes induced by miR-34a was observed by phase-contrast microscopy, resulting in a slight inhibition of miR-34a-induced morphological changes at 72 h, but not at 24 h (Figure S4). Additionally, SB suppressed miR-34a-induced alterations in mRNA expression of α-SMA and vimentin at 72 h, but not at 24 h (Figure 6A), which was comparable to the upregulation of p-SMAD2 by the miR-34a mimic at 72 h, but not at 24 h, as shown in Figure 6B. Notably, SB had no effect on miR-34a-induced upregulation of p53 protein level, whereas the increase in protein expression of α-SMA by miR-34a mimic was reversed by co-treatment with SB (Figure 6B). Notably, under p53-knockdown conditions, miR-34a-induced upregulation of p-SMAD2 at 72 h was not observed (Figure 6C), indicating that the SMAD2 pathway is associated with p53-mediated regulation of EMT induced by miR-34a.

## 4. Discussion

As a clinical study of microRNA-based cancer therapy using a liposomal mimic of miR-34a is proceeding to achieve the first clinical use of miRNA [4], the tumor-suppressive effect of miR-34a has been well investigated. Using A549 cells as NSCLC, it has been reported that miR-34a works as a tumor suppressor by targeting SIRT6 [19]. In the present study, we found that miR-34a clearly promoted the apoptosis ratio in A549 cells, whereas the apoptosis ratio in miR-34a mimic-transfected H1299 cells was lower than that in A549 cells, indicating that p53 influences miR-34a-induced apoptosis. Conversely, miR-34a transfection for 72 h led to a slight increase in the apoptosis ratio in H1299 cells, which was comparable to the finding that miR-34a promoted the apoptosis of H1299 cells [20]. The difference in p53 expression between A549 and H1299 cells affects the sensitivity of chemotherapeutic agents as well as miR-34a, as evidenced by the antiproliferative effect of gefitinib being more sensitive in A549 cells (IC_50_, 5 µM) than in H1299 cells (IC_50_, 40 µM) [21]. Thus, the tumor-suppressive effect of miR-34a in NSCLC may partly depend on p53 activity, which is a critical point in the clinical use of miR-34a.

Our previous work revealed that MTX independently induced apoptosis and EMT in A549 cells, and MTX-induced EMT corresponded well with the p53/p21 axis [22]. In the type II alveolar epithelium model cell line, A549/ABCA3 was modified from wild-type A549, and we found that chemotherapeutic drugs, such as bleomycin (BLM) and MTX, led to EMT via the activation of the p53/miR-34a axis [10]. These findings led to the hypothesis that p53 contributes to miR-34a-induced EMT. Therefore, the effect of miR-34a overexpression on EMT-like phenotypes in p53-wild type A549 cells and p53-null H1299 cells was examined. As expected, miR-34a mimic-induced morphological changes, expression alterations of EMT-related genes such as α-SMA and vimentin, and migration rate were more sensitive in A549 cells than in H1299 cells. In parallel with these observations, miR-34a led to p53 activation and upregulated p53 protein and CDKN1A mRNA expression. Regarding the transcriptional factors related to EMT process, mRNA expression level of SLUG was clearly increased by miR-34a mimic transfection in A549 cells, but not in H1299 cells. However, these miR-34a-induced EMT-like phenotypic changes were limited to changes in α-SMA expression under p53-knockdown conditions, supporting the novel idea that p53 specifically contributes to the upregulation of α-SMA in miR-34a-induced EMT.

Recently, several reports have suggested that EMT is associated with resistance to apoptosis [23,24], which may not be comparable to our findings that miR-34a induced both apoptosis and EMT in A549 cells. Conversely, in A549 cells, we previously confirmed that MTX induced both apoptosis and EMT under the same conditions, which is due to the heterogeneous features of A549 cells, as evidenced by clone A549 cells with different responses to MTX treatment and no relative correlation of MTX-induced apoptosis with EMT [22]. Therefore, the apoptosis pathway is independent of the EMT process under MTX treatment in A549 cells. In H1299 cells, in the present study, miR-34a tended to increase apoptosis in A549 cells at 72 h, suggesting that miR-34a induces apoptosis without p53 activity. Accordingly, miR-34a-induced apoptosis and EMT are also independent, and p53 may be partly involved in miR-34a-induced apoptosis via different mechanisms from EMT. However, the underlying mechanism of EMT-related resistance against apoptosis is poorly understood. Further studies are needed to clarify the relationship between miR-34a-induced apoptosis and EMT in A549 cells.

It is well known that α-SMA is an isoform of actin that is positive in myofibroblasts and is a representative EMT marker. Additionally, α-SMA is currently recognized as a promising prognostic biomarker and/or therapeutic target for metastatic lung and ovarian cancers [25,26]. Thus, the expression of α-SMA is closely associated with cancer progression. In contrast, p53 is known to transcriptionally regulate α-SMA expression [27], supporting the positive relationship between p53 activity and mRNA/protein expression levels of α-SMA observed in the present study. In our opinion, the present results, especially the upregulating effect of miR-34a on the expression level of α-SMA in A549 cells, would lead to concerns regarding the clinical use of miR-34a preparations in p53-positive tumor cells.

In contrast to α-SMA, mRNA expression of vimentin and SLUG, and migration rate enhanced by miR-34a mimic transfection was not affected by knocking p53 in A549 cells, while miR-34a-induced upregulation of vimentin, SLUG, and migration rate were not observed in H1299 cells. These findings suggest that factors other than p53 may determine the sensitivity of A549 and H1299 cells to miR-34a, whereas further studies are needed to clarify the crucial factors that specify the differences between both cells. Meanwhile, it has been reported that vimentin expression and migrative activity are originally enhanced in H1299 cells compared with A549 cells [28,29], and A549 cells showed greater invasiveness than H1299 cells under treatment with the well-known EMT inducer TGF-β1 [30], which may be considered as a cause of the lower sensitivity of H1299 cells to miR-34a. SLUG is known as an invasion promoter by repressing E-cadherin transcription, and p53 contributes to maintaining E-cadherin by enhancing the degradation of SLUG by forming MDM2-p53-SLUG complex [31]. However, MDM2 also stabilizes SLUG mRNA in lung cancer cell lines [32]. Thus, a complex interaction of p53 with SLUG mediated by MDM2 is proposed, but there is no effective information to construct the speculated mechanism of p53-independent miR-34a-induced upregulation of SLUG in only A549 cells.

The TGF-β signaling pathway has been shown to contribute to the development of pathophysiological conditions such as cancer progression [33] and organ fibrosis [34], and several miRNAs, including miR-34a, are involved in the regulation of the TGF-β signaling pathway [35]. In the present study, miR-34a promoted phosphorylation of SMAD2 in A549 cells, which corresponds to a previous finding that miR-34a mimic overexpression in human intrahepatic biliary epithelial cells leads to enhancement of p-SMAD2 [13]. Additionally, SB, an inhibitor of the TGF-β signaling pathway, suppressed increase in both expression level of α-SMA and p-SMAD2 enhanced by miR-34a, indicating that miR-34a-induced EMT in A549 cells is mediated by the TGF-β signaling pathway. Notably, it is of great interest that miR-34a mimic transfection for 24 h had no effect on the phosphorylation of SMAD2, and p53-knockdown canceled the miR-34a-induced increase in p-SMAD2 in 72 h, which assumes that p53 activation by miR-34a in the early phase may enhance the TGF-β signaling pathway in the late phase.

An increasing number of reports have documented the suppressive effect of miR-34a on tumor cell population, metastasis, and invasiveness, suggesting a significant association between miR-34a and tumor-associated EMT [36,37]. In fact, miR-34a negatively regulates EMT-associated transcriptional factors, such as SNAIL, SLUG, ZEB1/2, and TWIST1, via its binding to the 3′-UTR of these factors [15]. Conversely, the present study showed the opposite finding that miR-34a promoted EMT. To resolve the discrepancy between miR-34a-mediated negative or positive regulation of EMT, direct targets of miR-34a for promoting EMT, such as SNAIL, SLUG, ZEB1/2, and TWIST1 in the case of miR-34a-induced suppression of EMT. It is difficult to determine the net contribution of miR-34a to the EMT process.

## 5. Conclusions

The present study aimed to characterize miR-34a-induced EMT in p53-wild type A549 and p53-null H1299 cells. We demonstrated that miR-34a induced EMT-like phenotypic changes, such as mRNA/protein expression alterations and enhanced migration efficacy, in A549 cells, but not in H1299 cells. Among the factors associated with miR-34a-induced EMT in A549 cells, only the α-SMA mRNA/protein expression levels increased by miR-34a were suppressed by p53-knockdown. These findings strongly indicate that miR-34a can induce EMT in p53-wild type NSCLCs, which may lead to further cancer progression mediated by EMT. Therefore, miR-34a-induced EMT should be highlighted under the clinical use of miR-34a preparations in the future, especially in p53-wild type cancer cell lines. The results obtained in the present study provide important insights about the effective use of miR-34a in clinical situations to avoid miR-34a-induced EMT.

## Figures and Tables

**Table 1:** Primer Sequences for real-time PCR analysis.

Name	Accession Number	Sequence
α-SMA	NC_000010.11	Foward: (5′)GCTGTTTCCCATCCATTGT(3′)
		Reverse: (5′)TTTGCTCTGTGCTTCGTCAC(3′)
CDKN1A	NC_000006.12	Foward: (5′)GTGGACCTGTCACTGTCTTG(3′)
		Reverse: (5′)GGCGTTTGGAGTGGTAGAAA(3′)
GAPDH	NC_000012.12	Foward: (5′)ACGGGAAGCTTGTCATCAAT(3′)
		Reverse: (5′)TGGACTCCACGACGTACTCA(3′)
N-cadherin	NC_000018.10	Foward: (5′)AATCAGTGGCGGAGATCCTA(3′)
		Reverse: (5′)CCTTGGCTAATGGCACTTGA(3′)
SNAIL	NC_000020.11	Foward: (5′)GAGTGGTTCTTCTGCGCTAC(3′)
		Reverse: (5′)TAGGGCTGCTGGAAGGTAAA(3′)
SLUG	NC_000008.11	Foward: (5′)TATTCGGACCCACACATTAC(3′)
		Reverse: (5′)CAGATTTGACCTGTCTGCAA(3′)
TWIST	NC_000007.14	Foward: (5′)AGCTATGTGGCTCACGAG(3′)
		Reverse: (5′)TGTCCATTTTCTCCTTCTCTGG(3′)
Vimentin	NC_000010.11	Foward: (5′)TCAGAGAGAGGAAGCCGAAA(3′)
		Reverse: (5′)CAAAAAGGCAATCTCTTCTTGC(3′)
ZEB1	NC_000010.11	Foward: (5′)AGCTGCCAATAAGCAAACGA(3′)
		Reverse: (5′)TTTGGGCGGTGTAGAATCAG(3′)

## Data Availability

Not applicable.

## Supplementary Materials

The following are available online at https://www.mdpi.com/article/10.3390/biom11121853/s1, Figure S1, Selection and efficacy of small interfering RNAs (siRNAs) for p53 in A549 cells; Figure S2, Morphologies of A549 and H1299 cells treated with the microRNA (miR)-34a mimic; Figure S3, Effect of miR-34a overexpression on epithelial–mesenchymal transition (EMT)-related phenotypical changes in A549 cells under p53-knockdown condition for 24 h; Figure S4, Morphologies of A549 cells treated with the miR-34a mimic in the absence or presence of SB431542 (SB) for 24 or 72 h.

## Abbreviations

epithelial–mesenchymal transition	EMT
microRNA	miRNA
non-small cell lung cancer	NSCLC
