# Supplementary material for: Characterization of miR-34a-Induced Epithelial–Mesenchymal Transition in Non-Small Lung Cancer Cells Focusing on p53
Source: Biomolecules. 2021 Dec 9;11(12):1853. doi: 10.3390/biom11121853 (PMC8699678; doi:10.3390/biom11121853)
Supplement: Supplementary file 1 [file biomolecules-11-01853-s001.zip › biomolecules-1469212-supplementary.pdf]

# Figure S1

(A)

| | siRNA sequence | Target (GGGenome) |
|---|---|---|
| #1 | 5'- GUUCAAGACAGAAGGGCCUtt<br>5'- AGGCCCUUCUGUCUUGAACtt | NM_001126114.2\|TP53\|chr17:7668401:7687550:-\|spliced<br>position: 1487-1505<br>1487<br>TCAGTCTACCTCCCGCCATAAAAAACTCATGTTCAAGACAGAAGGGCCTGACTCAGACTGACATTCTCCACTTCTTGTT |
| #2 | 5'- GUGAGGGAUGUUUGGGAGAtt<br>5'- UCUCCCAAACAUCCCUCACtt | NM_001126114.2\|TP53\|chr17:7668401:7687550:-\|spliced<br>position: 1755-1773<br>1755<br>CATACCAGCTTAGATTTTAAGGTTTTTACTGTGAGGGATGTTTGGGAGATGTAAGAAATGTTCTTGCAGTTAAGGGTTA |
| #3 | 5'- CCUUAGUACCUAAAAGGAAtt<br>5'- UUCCUUUUAGGUACUAAGGtt | NM_001126114.2\|TP53\|chr17:7668401:7687550:-\|spliced<br>position: 2154-2172<br>2154<br>CCAAACCCTGTCTGACAACCTCTTGGTGAACCTTAGTACCTAAAAGGAAATCTCACCCCATCCCACACCCTGGAGGATT |

(B)

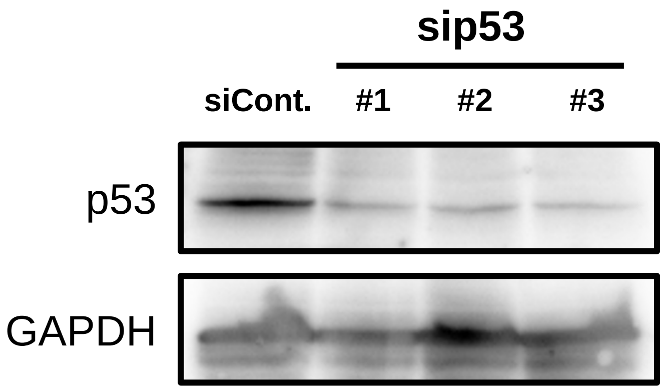


(C)

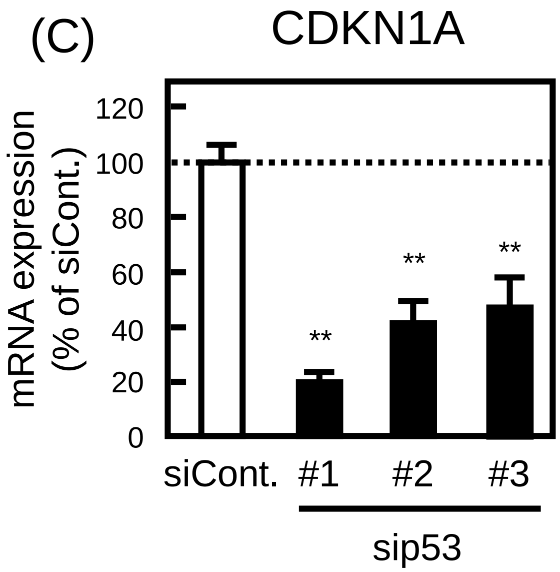


Figure S1. Selection and efficacy of small interfering RNAs (siRNAs) for p53 (#1-3) in A549 cells. (A) Sequences for sip53 #1-3 and its target region searched by GGGenome (https://gggenome.dbcls.jp) are listed. The cells were transfected with control siRNA (siCont.; 10 pmol/well) or sip53 #1-3 (10 pmol/well) for 24 h. After 72 h, (B) protein expression of p53 was detected by western blot, and (C) mRNA expression of CDKN1A was analyzed by real-time PCR. Each value represents the mean ± S.E.M. (n=3). **p<0.01: significantly different from NC.

# Figure S2

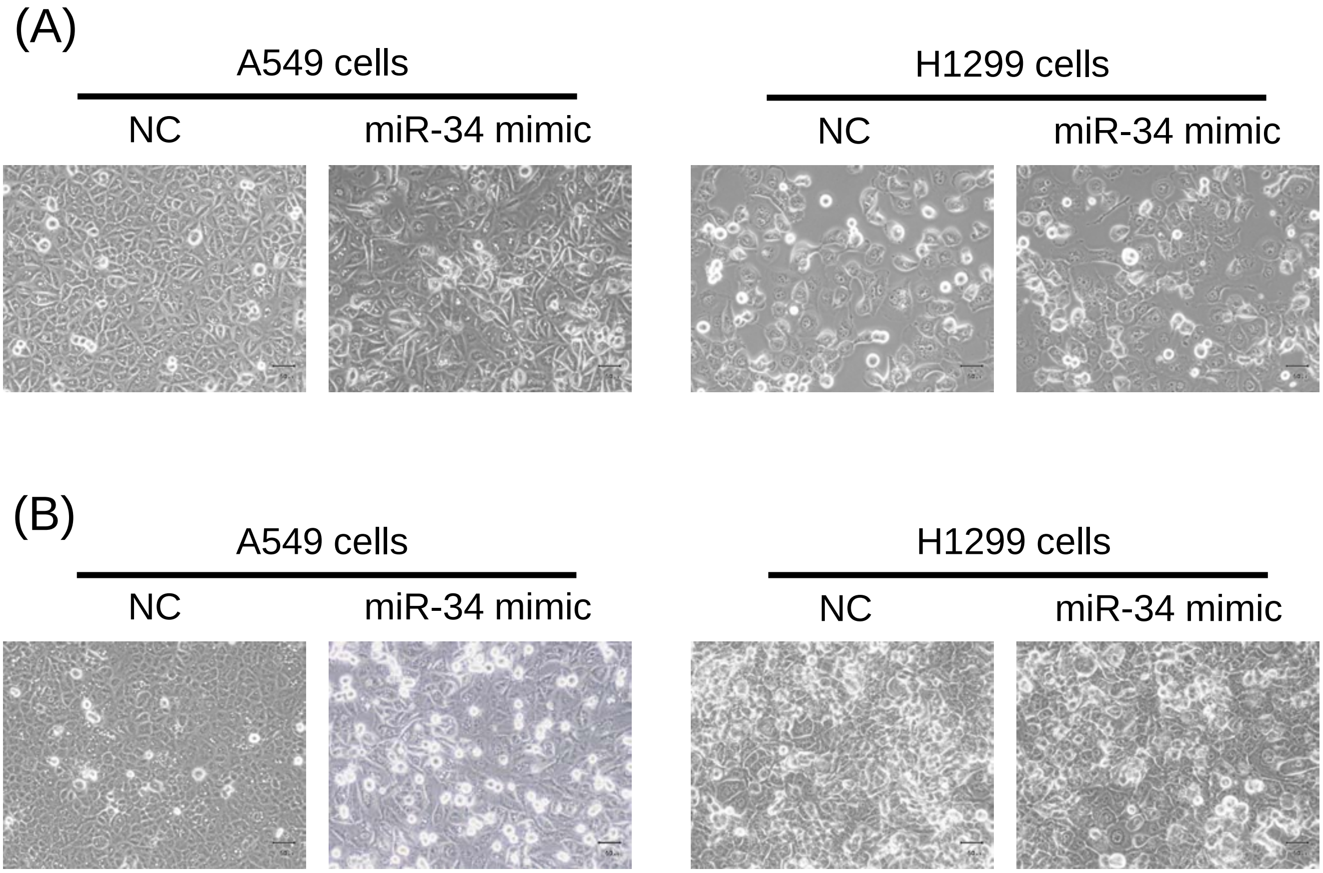


Figure S2. Confirmation on morphology of A549 and H1299 cells treated with miR-34a mimic. These cells were transfected with negative control (NC; 20 pmol/well) or miR-34a mimic (20 pmol/well) for 24 h. After 24 (A) or 72 h (B), the morphology of the cells was observed by phase-contrast microscopy.

# Figure S3

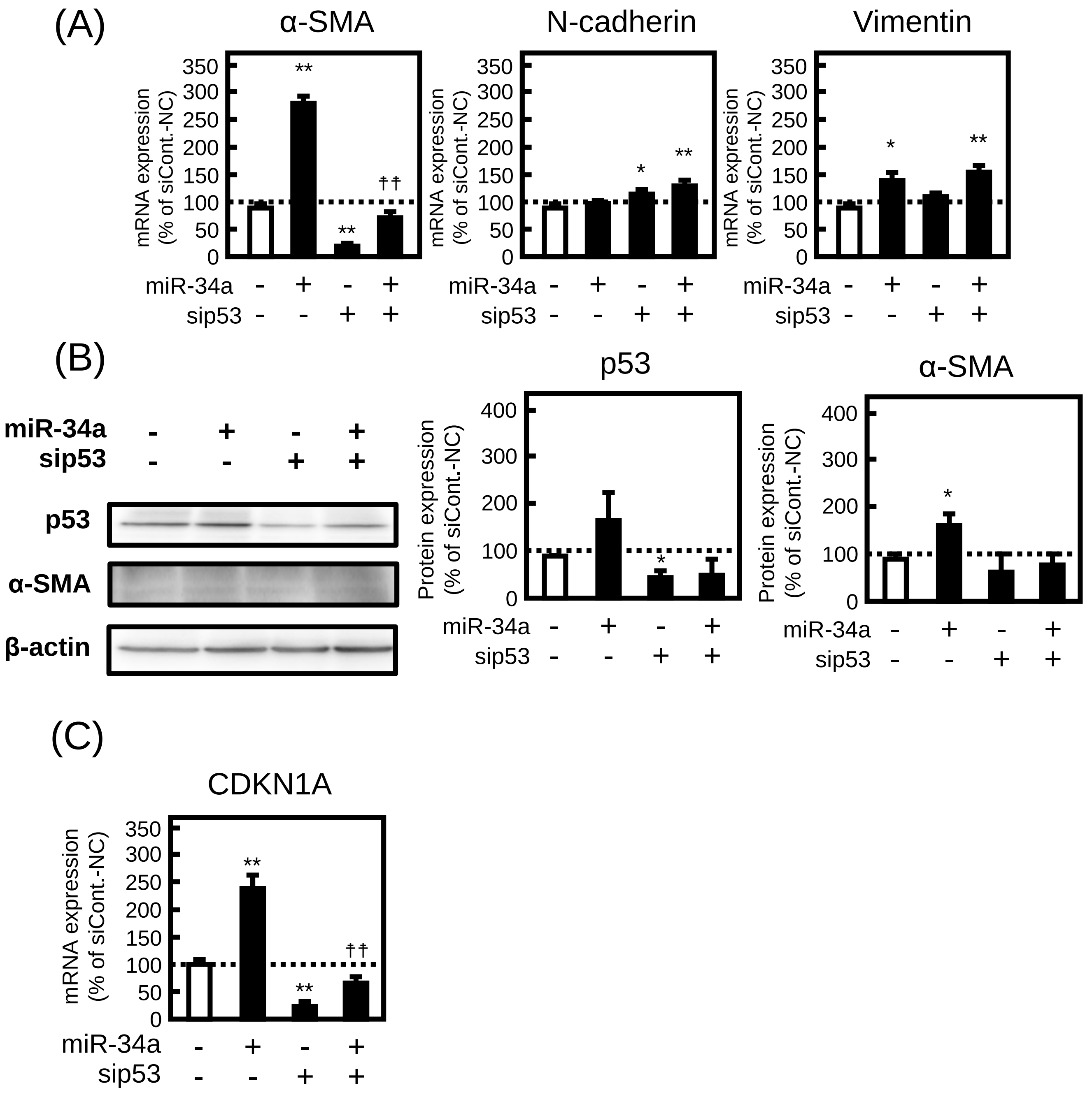


Figure S3. Contribution of p53 to EMT-related phenotypes in A549 cells under the miR-34a overexpression. The cells were transfected with negative control (NC; 20 pmol/well) or miR-34a mimic (20 pmol/well) under the control siRNA (siCont.; 10 pmol/well) or sip53 (10 pmol/well) for 24 h. After 72 h, mRNA expression levels of (A) α-SMA, Vimentin, N-cadherin, and (C) CDKN1A were measured by real-time PCR. (B) The protein level of α-SMA and p53 were analyzed by Western blot. Each value represents the mean ± S.E.M. (n=3). *$p<0.05$, **$p<0.01$: significantly different from NC under siCont. condition, ††$p<0.01$: significantly different from miR-34a mimic under siCont. condition.

# Figure S4

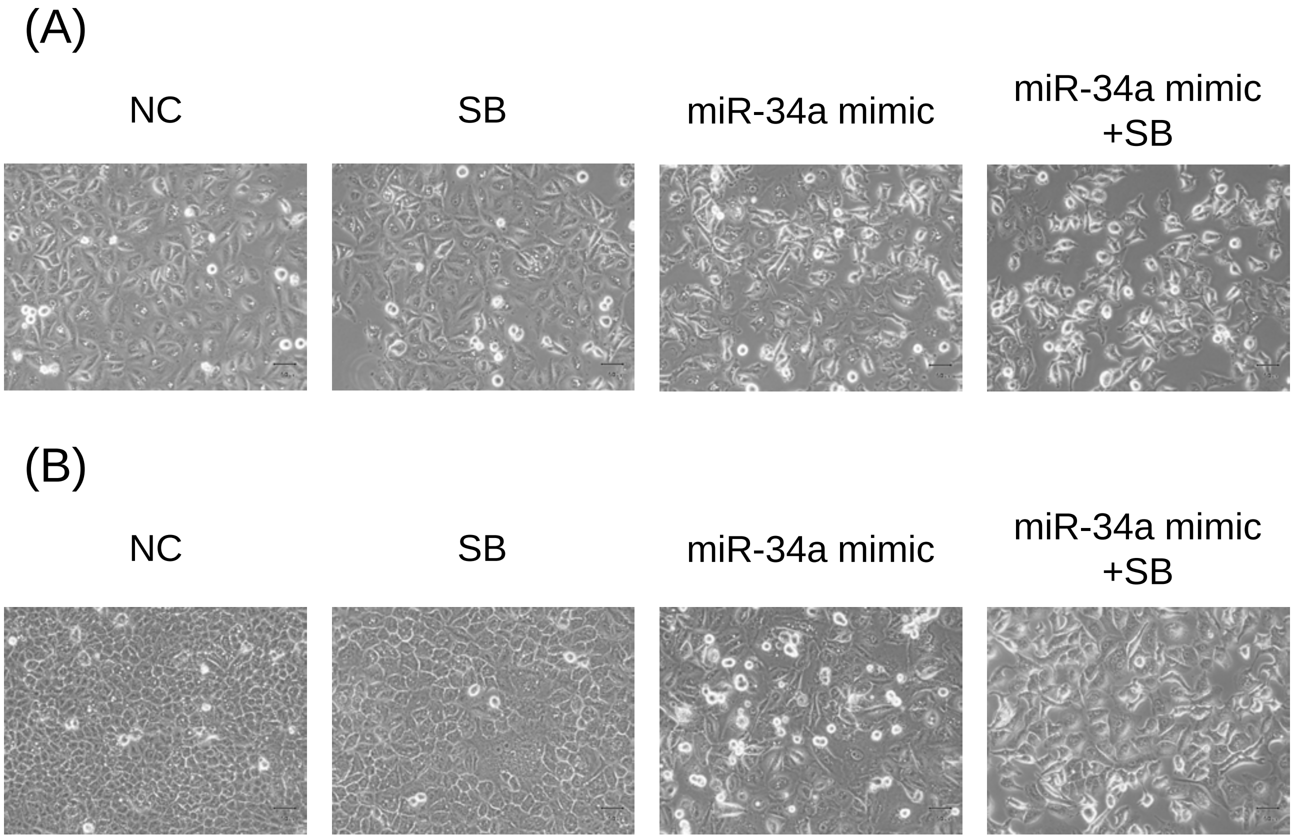


Figure S4. Contribution of TGF-β/SMAD signaling to miR-34a-induced changes in morphology of A549 cells. The cells were transfected with negative control (NC; 20 pmol/well) or miR-34a mimic (20 pmol/well) for 24 h. After removal of the medium containing miRNA mimic, the cells were treated with or without 10 μM SB43142 (SB) for 24 (A) or 72 h (B). The morphology of the cells was observed by phase-contrast microscopy.
